# Efficacy of Actellic 300 CS-based indoor residual spraying on key entomological indicators of malaria transmission in Alibori and Donga, two regions of northern Benin

**DOI:** 10.1186/s13071-019-3865-1

**Published:** 2019-12-30

**Authors:** Albert Sourou Salako, Fortune Dagnon, Arthur Sovi, Gil Germain Padonou, Rock Aïkpon, Idelphonse Ahogni, Thomas Syme, Renaud Govoétchan, Herman Sagbohan, André Aimé Sominahouin, Bruno Akinro, Laurent Iyikirenga, Fiacre Agossa, Martin Codjo Akogbeto

**Affiliations:** 1 Centre de Recherche Entomologique de Cotonou (CREC), Cotonou, Benin; 2 Faculté des Sciences et Techniques de l’Université d’Abomey-Calavi, Cotonou, Benin; 3 USA President’s Malaria Initiative, USA Agency for International Development, Cotonou, Benin; 4 Faculty of Agronomy, University of Parakou, BP 123, Parakou, Benin; 5 Disease Control Department, Faculty of Infectious & Tropical Diseases, The London School of Hygiene and Tropical Medicine, Keppel Street, London, WC1E 7HT UK; 6 Université Nationale des Sciences, Technologies, Ingénierie et Mathématiques, Abomey, Bénin; 7 Faculté des Sciences Humaines et Sociales de l’Université d’Abomey-Calavi, Abomey-Calavi, Benin; 8 PMI VectorLink Project, Abt Associates, Cotonou, Benin; 9 PMI VectorLink Project, Abt Associates, Kinshasa, Democratic Republic of Congo

**Keywords:** IRS, Efficacy, Actellic 300CS, Malaria transmission, Benin

## Abstract

**Background:**

The current study shows the results of three years of IRS entomological monitoring (2016, before intervention; 2017 and 2018, after intervention) performed in Alibori and Donga, northern Benin.

**Methods:**

Mosquito collections were performed on a monthly basis using human landing catches and pyrethrum spray catches in six districts including four treated with Actellic 300 CS (Kandi, Gogounou, Djougou and Copargo) and two untreated (Bembèrèkè and Kouandé) which served as control sites. Key transmission indicators of *Anopheles gambiae* (*s.l.*) as well as the residual activity of Actellic 300 CS assessed through WHO cone tests, were determined.

**Results:**

The residual efficacy duration of Actellic 300 CS after the two IRS campaigns (2017 and 2018) was 4–5 months (May–September). The parity rate and the sporozoite index of *An. gambiae* (*s.l.*) were 36.62% and 0.71%, respectively, after the first spray round in treated areas compared to 57.24% and 3.7%, respectively, in the control areas (*P* < 0.0001). The same trend was observed after the second spray round. After the first spray round, each person received 1.6 infective bites/month (ib/m) in the treated areas against 12.11 ib/m in the control areas, resulting in a reduction rate of 86.78%. Similarly, the entomological inoculation rate was 1.5 ib/m after the second spray round in the treated areas *vs* 9.75 ib/m in the control areas, corresponding to a reduction of 84.61%. A decrease in the parity rate (46.26%), sporozoite index (85.75%) and EIR (87.27%) was observed for *An. gambiae* (*s.l*.) after the first round of IRS (June–October 2017) compared to the pre-intervention period (June–October 2016). The density of *An. gambiae* (*s.l*.) ranged between 0.38–0.48 per house in treated areas *vs* 1.53–1.76 *An. gambiae* (*s.l*.) per house respectively after the first and second IRS rounds.

**Conclusions:**

This study showed the positive impact of IRS in reducing key entomological parameters of malaria transmission in Alibori and Donga. However, the considerable blood-feeding rate of *An. gambiae* (*s.l*.) in spray areas, stress the need for the population to sleep under long-lasting insecticidal nets (LLINs) in addition, to prevent from mosquito bites which did not succeed in resting on sprayed walls.

## Background

Over the past decade, progress has been made in malaria control, through the promotion of indoor residual spraying (IRS) and long-lasting insecticidal nets (LLINs) [1–5]. Indeed, the proportion of the population with access to LLINs or having benefited from IRS in sub-Saharan Africa increased significantly from 2% in 2000 to 59% in 2014 [5]. According to the World Health Organization (WHO), of the 663 million malaria cases prevented in sub-Saharan Africa between 2001 and 2015 through vector control interventions, 79% were through LLINs and IRS [5]. This historic progress in the fight against malaria is partly due to the efforts of the USA Government through the Presidentʼs Malaria Initiative (PMI) [6].

In Benin, IRS to control malaria vectors was introduced due to the expansion of pyrethroids resistance [7–10] which was the only insecticide class approved for the impregnation of LLINs. The strategy first implemented with bendiocarb (2008–2010) in the Oueme region (south Benin) and then, transferred to the Atacora region (north Benin) with the use of the same product (2011–2013) later replaced by pirimiphos-methyl (2014–2016), was very successful [11–13]. Indeed, Atacora region offers a good cost-effectiveness ratio insofar as a single IRS round is sufficient to cover its short transmission period, which favored the relocation of the intervention to that region. In addition, the decrease in susceptibility of mosquitoes to bendiocarb in the same region [14] has favored the switch from this insecticide to pirimiphos-methyl, which showed good performance in experimental huts trials [15].

After six years of IRS implementation in the Atacora region, the National Malaria Control Programme (NMCP), in agreement with various partners, decided to partially withdraw the intervention from some districts of this region and relocate it to two other regions (Alibori and Donga). This decision not only falls within the framework of the implementation of the national insecticide resistance management plan, but provides opportunity for coverage in two high burden regions [16] which have never benefitted from this intervention. In preparation for this relocation, prior studies have shown that *An. gambiae* (*s.l.*), the main malaria vector in the two target regions [17, 18], was susceptible to pirimiphos-methyl [19, 20], an organophosphate insecticide and potential candidate for the IRS campaigns in Alibori and Donga.

In 2017 and 2018, all houses in Djougou, Copargo and Ouake (Donga region) and Kandi, Gogounou and Segbana districts in the Alibori region were treated with Actellic 300CS (pirimiphos-methyl). The present study shows the results of the IRS entomological monitoring conducted in both regions. Thus, the impact of the strategy on key entomological indicators of malaria transmission as well as, the residual efficacy duration of Actellic 300CS on the different type of sprayed walls were evaluated.

## Methods

### Study area

**Fig. 1:**
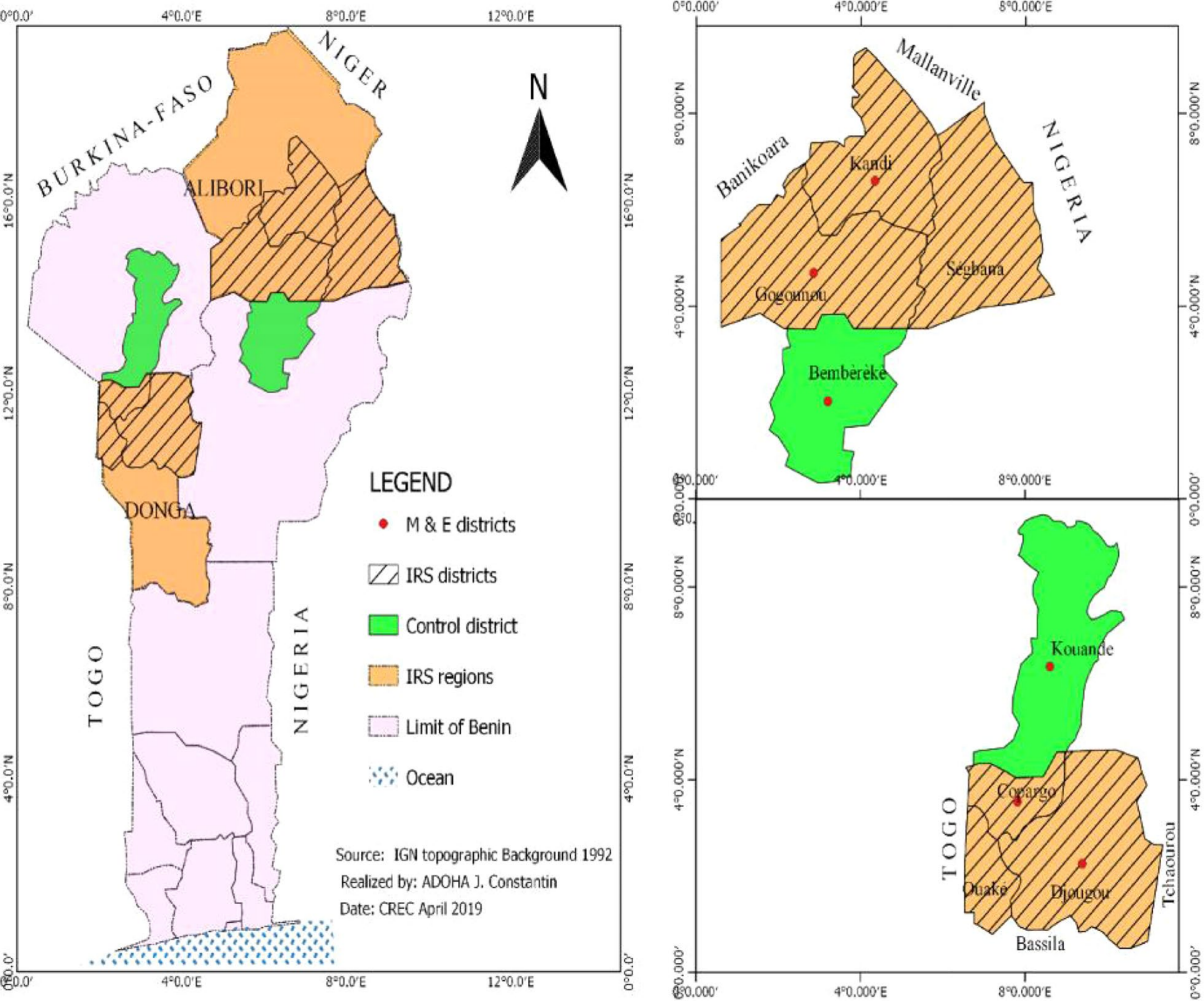
Map of study area located within the northern region of Benin

In 2017, Benin’s NMCP relocated IRS to six districts of northern Benin, including three districts (Kandi, Gogounou and Segbana) in the Alibori region and three other (Djougou, Copargo, Ouake) in the Donga region (Fig. 1). A total of 1,226,161 and 1,287,469 persons were protected with Actellic 300 CS (pirimiphos-methyl) respectively in 2017 and 2018. For the entomological monitoring of the intervention, 4 districts were surveyed including Djougou (09°42′10″N, 01°40′55″W) and Copargo (09°50′19″N, 01°32′39″W) in northwest of Benin and, Kandi (11°07′29″N, 2°56′9″W) and Gogounou (10°50′30″N, 2°50′20″W) in northeast of the country (Fig. 1). Adjacent districts served as controls including Kouande (10°19′54″N, 1°41′29″W) which is close to the IRS districts of Djougou and Copargo and, Bembereke (10°13′30″N, 02°40′05″W) that is next to the IRS districts of Kandi and, Gogounou (Fig. 1).

The climate is Sudano-Guinean in the Donga region and Sudanese in the Alibori region. These two regions are dry savannah areas, with six months rainy season (mid-April to mid-October) and a dry season which spans the remainder of the year. Overall, average annual rainfall ranges between 700–1200 mm and 1200–1300 in Alibori and Donga, respectively.

The incidence of simple and severe malaria in 2016 was 26.4% in Donga region and 13.3% in Alibori [16].

### Adult mosquito collections

In each evaluation district, a central site and a peripheral site were selected. On each site, human landing catches (HLC) were conducted from 21:00 h to 5:00 h in 2 houses randomly selected at each field visit, with one collector sitting indoor and a second outdoor, amounting to a total of 4 collectors/hour/site and 8 collectors/hour/district over each sampling night. On each site, the 4 persons who collected the mosquitoes from 21:00 h to 1:00 h were replaced by 4 other collectors from 1:00 h to 5:00 h. They were rotated between the different houses to avoid bias related to their ability to capture mosquitoes or, their individual attractiveness. This sampling method allowed to evaluate the human-biting rate (HBR) of the *Anopheles* vectors, which were then analyzed by ELISA circumsporozoite protein (CSP) to determine their sporozoite index (SI). The morning collection (from 6:00 h to 7:00 h) of indoor resting mosquitoes using pyrethrum spray catch (PSC) was carried out in 20 houses per district (10 selected from each central and peripheral site). This sampling technique allowed us to estimate the mean indoor vector density.

### Mosquito identification and processing

#### Morphological identification of vectors species

After each collection, mosquitoes were counted and morphologically identified using the taxonomic key of Gillies & Meillon [21]. About 40–50% of the *Anopheles* vectors captured through HLC were dissected to assess their physiological age [22]. Those collected by PSC were classified according to the physiological state (unfed, feed, half-gravid and gravid) of their abdomen. Each specimen was then stored in a labeled Eppendorf tube containing silica gel and cotton for further molecular analyses.

#### Molecular analyses

To detect the presence of *P. falciparum*, heads and thoraces of all females *An. gambiae* (*s.l*.) were analyzed by ELISA CSP according to the protocol described by Wirtz et al. [23]. The abdomens, legs and wings of 20 to 50 specimens of *An. gambiae* (*s.l*.), randomly selected in each district each month, were analyzed by PCR according to the protocol of Santolamazza et al. [24], for molecular species identification.

### WHO cone bioassays

The residual activity of Actellic 300 CS on treated walls was evaluated after each IRS campaign using WHO cone bioassays [25]. These bioassays were carried out with females of *An. gambiae* Kisumu, a laboratory susceptible strain reared and maintained at the Centre de Recherche Entomologique de Cotonou (CREC).

#### Cone bioassay procedure

From 2017 to 2018, monthly cone bioassays [T0 (May), T1 (June), T2 (July), T2 (July), T3 (August), T4 (September), T5 (October) and T6 (November)] were conducted on treated walls of 20 houses randomly selected in the Donga and Alibori regions. The surfaces of the untreated walls were used as a control. These bioassays aimed not only to evaluate the quality of the treatment applications but also to monitor the residual effect of Actellic 300 CS on the treated walls. The bioassays were performed on the cement and mud walls encountered in the study area. Using a mouth aspirator, 15 females *An. gambiae* Kisumu aged 2–5 days-old were carefully introduced into each cone, fixed at three different heights (0.5 m, 1 m and 1.5 m) of the treated walls. Mosquitoes were exposed to the sprayed walls for 30 min; then removed from the cones and transferred to labeled sterile cups and provided with 10% sugar solution. After 24 h of observation at a temperature of 27 ± 2 °C and a relative humidity of 80 ± 10%, the mortality rate was determined. When the control mortality was between 5–20%, corrected mortality was performed accordingly using Abottʼs formula [26]; when the control mortality was higher than 20%, the bioassay was considered invalid and repeated.

### Estimation of entomological parameters

In this study, entomological parameters measured before (May 2016–April 2017) and after IRS (June 2017–August 2018) include: (i) human-biting rate (HBR), number of bites of *An. gambiae* (*s.l*.) per unit of time (HBR = No. of specimens of *An. gambiae* (*s.l*.) collected/No. of collectors/No. of nights of sampling); (ii) sporozoite index (SI), proportion of *An. gambiae* (*s.l*.) with circumsporozoite protein of *P. falciparum* (SI = (No. of positive thoraces/Total no. of analyzed thoraces) × 100); (iii) parity rate (PR), percentage of parous *An. gambiae* (*s.l*.) (PR = (No. of parous mosquitoes/Total no. of dissected mosquitoes) × 100); (iv) indoor vector density (IVD), mean number of *An. gambiae* (*s.l*.) collected per house (Total no. of *An. gambiae* (*s.l*.) collected indoors by PSC/Total no. of surveyed houses); (v) blood-feeding rate, proportion of *An. gambiae* (*s.l*.) having blood-fed [BFR = (No. of blood-fed and half-gravid vectors collected by PSC/Total no. of vectors collected by the same method) × 100]; (vi) entomological inoculation rate (EIR), level of malaria transmission by *An. gambiae* (*s.l*.) (EIR = HBR × SI).

### Data analysis

Data were analyzed with the statistical R software, version 2.8. using the *stats* package [27]. The Poisson method was used to estimate and compare the confidence intervals of indoor vector density and EIRs of *An. gambiae* (*s.l*.) [28]. The Chi-square test of comparison of proportions was used to compare blood-feeding rate, sporozoite index, and parity rate of *An. gambiae* (*s.l*.). These different parameters were compared before and after IRS and then between the treated and control areas.

## Results

### Residual effect of Actellic 300 CS on treated walls from 2017 and 2018

**Fig. 2:**
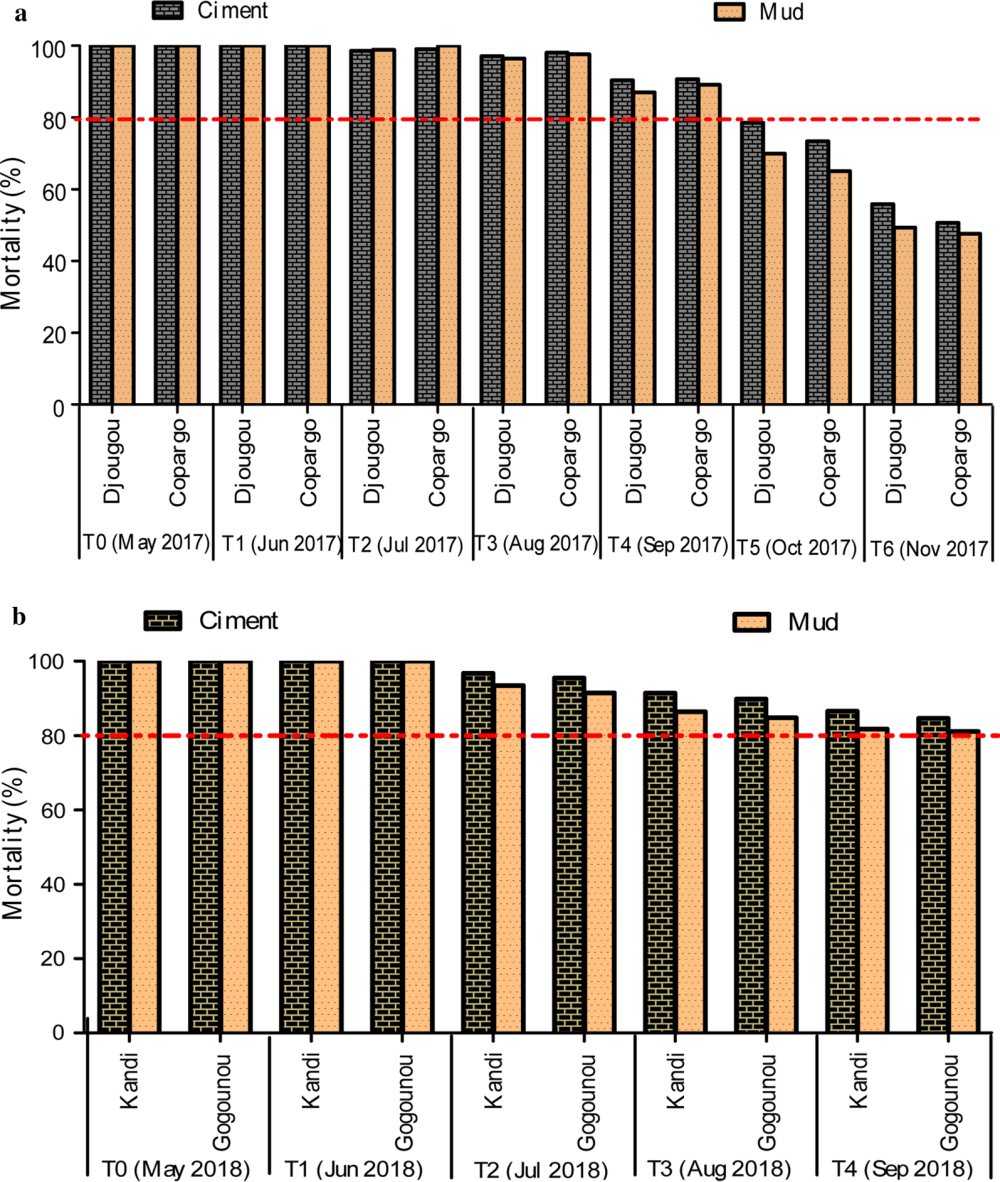
Mortality rate of *Anopheles gambiae* Kisumu (laboratory susceptible strain) after 30 min exposure to cement and mud walls treated with pirimiphos-methyl in 2017 (**a**) and 2018 (**b**). The red line indicates the WHO efficacy threshold (mortality of 80%) of an insecticide

Figure 2 shows the monthly variation of mortality rates in 2017 (Fig. 2a) and 2018 (Fig. 2b), after exposure of *An. gambiae* Kisumu to cement and mud walls sprayed with Actellic 300CS in the districts of Djougou and Copargo. In 2017 and 2018, cone bioassays revealed full susceptibility (100% mortality) of *An. gambiae* Kisumu to all sprayed walls (cement and mud), one-week post-IRS intervention (Fig. 2a, b). Overall, monthly data collected in 2017 and 2018 showed mortality rates of ≥ 80% (WHO efficacy threshold) between May and September/October regardless the district or the type of tested wall, giving an efficacy duration of 4–5 months (Fig. 2a, b).

### Vector species composition

A total of 8776 *Anopheles* specimens belonging to seven species were collected through HLC over the study period in Alibori and Donga (treated areas from 2017), as well as in Bembereke and Kouande (control areas). *Anopheles gambiae* (*s.l*.) (98.05%, 8605/8776) was the most abundant species found, followed by *An. funestus* (1.59%, 140/8776). Only 15 *An. pharoensis* (0.17%), 9 *An. coustani* (0.10%), 5 *An. ziemanni* (0.05%), 1 *An. paludis* and 1 *An. nili* (0.01%) were collected (Table 1).

**Table 1:** *Anopheles* species composition in surveyed areas before and after IRS

Species	Before IRS (May 2016–April 2017)	After 1st round of IRS (June 2017–March 2018)	Control (Bembereke, Kouande)	After 2nd round of IRS (June 2018–November 2018)	Control (Bembereke–Kouande)	Total
*An. gambiae* (*s.l*.)	2465	2379	1546	1286	929	8605
*An. funestus*	54	54	16	9	7	140
*An. coustani*	9	0	0	0	0	9
*An. pharoensis*	3	2	5	2	3	15
*An. paludis*	1	0	0	0	0	1
*An. nili*	0	0	0	1	0	1
*An. ziemanni*	2	2	0	1	0	5
Total	2534	2437	1567	1299	939	8776

**Fig. 3:**
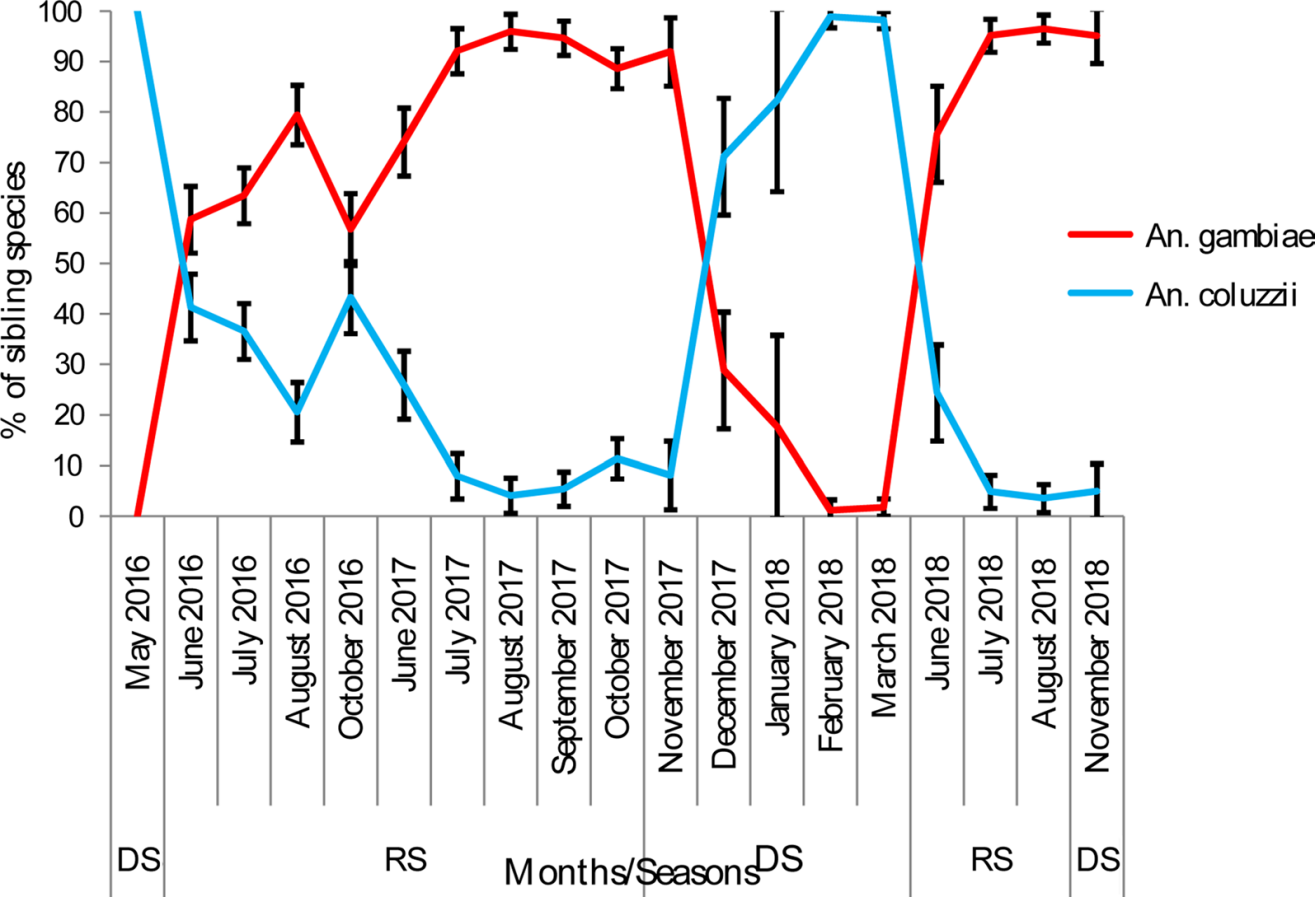
Seasonal variation of sibling species (*An. coluzzii* and *An. gambiae*) in the study area. *Abbreviations*: DS, dry season; RS, rainy season

Of the 2774 specimens of *An. gambiae* (*s.l*.) analyzed by PCR over the whole study period, three sibling species [*An. gambiae* (65.60%, *n* = 1820), *An. coluzzii* (33.42%, *n* = 927) and *An. arabiensis* (0.98%, *n* = 27)] were detected (Table 2). Overall, the same trend (predominance of *An. gambiae*) was observed before and post-IRS in all localities (treated and control) (Table 2). Seasonal variation in the frequency of *An. gambiae* and *An. coluzzii* was observed during the study (Fig. 3). Overall, out of a total of 628 mosquito specimens analyzed in the dry season, 77.7% (*n* = 488) of *An. coluzzii* were detected *vs* 22.3% (*n* = 140) of *An. gambiae*. In contrast, in the rainy season, *An. gambiae* was predominant (79.28%, 1680/2119) compared to *An. coluzzii* (20.72%, 439/2119) (Fig. 3).

**Table 2:** Frequency of sibling species of the *An. gambiae* (*s.l.*) complex in IRS and control areas

Period	No. of analysed samples in IRS areas	Alibori (IRS Area 1)			Donga (IRS Area 2)			Total (IRS Areas)			Bembereke (Control area)		
		*An. gambiae*	*An. coluzzii*	*An. arabiensis*	*An. gambiae*	*An. coluzzii*	*An. arabiensis*	*An. gambiae*	*An. coluzzii*	*An. arabiensis*	*An. gambiae*	*An. coluzzii*	*An. arabiensis*
Before IRS (May 2016–October 2016)	980	170	271	0	387	152	0	557	423	0	–	–	–
After IRS (June 2017–November 2018)	1794	466	176	17	520	147	5	986	323	22	277	181	5
Proportion (%)	–	70.71	26.70	2.57	77.38	21.87	0.74	74.07	24.26	1.65	59.82	39.09	1.07
Total (Before + after IRS)	2774	636	447	17	907	299	5	1543	746	22	277	181	5
Proportion (%)	–	57.81	40.63	1.54	74.89	24.69	0.41	66.76	32.28	0.95	59.82	39.09	1.07

### Impact of IRS on the longevity of *An. gambiae* (*s.l*.)

**Fig. 4:**
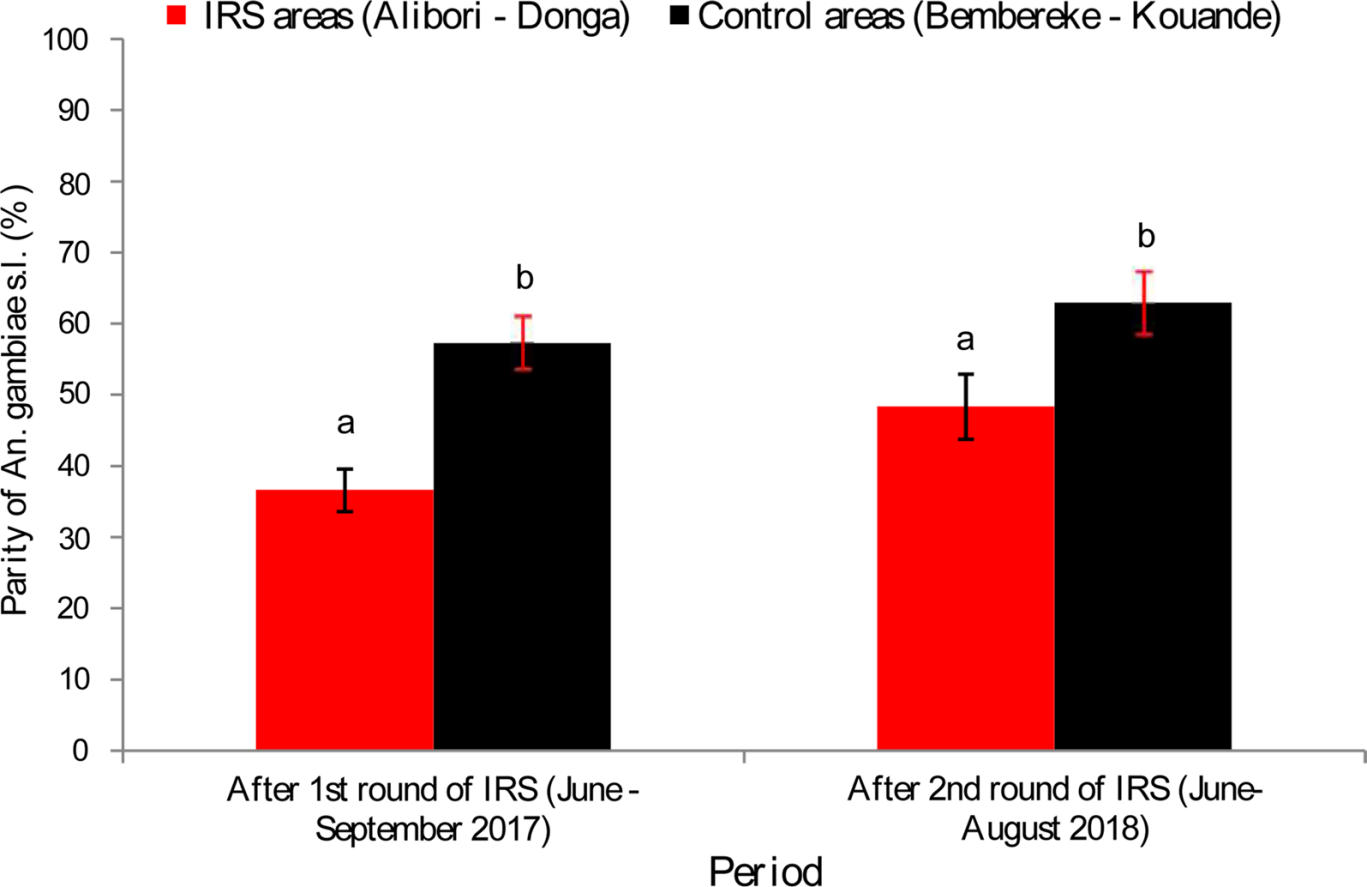
Parity rate of *Anopheles gambiae* (*s.l.*) collected in treated and control areas

Overall, Table 3 and Fig. 4 show the impact of IRS on the longevity of *An. gambiae* (*s.l*.) The parity rate of *An. gambiae* (*s.l*.) was 70.04% (996/1422) before IRS and 37.64% (457/1214) after the first round of IRS in Alibori and Donga, equating to a 46.25% reduction in vector longevity (*P* < 0.0001) (Table 3).

**Table 3:** Parity rates of *Anopheles gambiae* (*s.l*.) collected before and after the first round IRS

IRS area	Variable	Before IRS (June–October 2016)	After 1st round of IRS (June–October 2017)	*χ*^2^-value	*P*-value
Alibori-Donga	No. dissected	1422	1214	–	–
	No. parous	996	457	–	–
	Parous (%)	70.04	37.64	276.57	< 0.0001

The trend was the same when comparing the parity rates obtained in the treated and control areas during the efficacy period of Actellic 300 CS. Indeed, between June and September, the parity rates of *An. gambiae (s.l*.) in the treated areas (Alibori and Donga) were 36.62% (364/994) in 2017 and 48.36% (353/730) in 2018 against 57.24% (257/449) and 62.96 (289/459) in the control areas (*χ*^2^ = 52.798, *df* = 1, *P* < 0.0001 for 2017; *χ*^2^ = 23.621, *df* = 1, *P* < 0.0001 for 2018) (Fig. 4).

### Impact of IRS on the biting location of *An. gambiae* (*s.l.*)

Before IRS, the biting rate of *An. gambiae* (*s.l.*) was higher indoors [9.03 bites per person per night (b/p/n)] than outdoors (7.83 b/p/n) (*P* = 0.00098, *RR*=1.153 (95% CI: 1.06–1.26). Similarly, the biting activity of *An. gambiae* (*s.l*.) was more pronounced indoors than outdoors in the control areas after both the first (13.04 b/p/n indoors *vs* 8.58 outdoors; *RR* = 1.52 (95% CI: 1.35–1.72), *P* < 0.0001) and the second IRS rounds (14.2 b/p/n indoors *vs* 7.83 outdoors; *RR* = 1.81 (95% CI: 1.58–2.09), *P* < 0.0001) (Table 4). However, the opposite trend was observed after the first (4.86 b/p/n indoors *vs* 10.27 outdoors; *RR* = 2.11 (95% CI: 1.91–2.34), *P* < 0.0001) and the second (7.49 b/p/n indoors *vs* 8.03 outdoors; *RR*=1.07 (95% CI: 0.96–1.2), *P* = 0.233) rounds of IRS in the areas targeted by the strategy (Table 4).

**Table 4:** Biting location of *An. gambiae* (*s.l*.) in IRS and control areas

Period	Area	Location	No. collected	Person/night	HBR (b/p/n)	*P*-value
Before IRS (June–October 2016)	Future IRS	Indoor	1156	128	9.03	0.00098
		Outdoor	1002	128	7.83	
After 1st IRS round (June–September 2017)	IRS	Indoor	544	112	4.86	< 0.0001
		Outdoor	1150	112	10.27	
	Control	Indoor	678	52	13.04	< 0.0001
		Outdoor	446	52	8.58	
After 2nd IRS round (June–August 2018)	IRS	Indoor	599	80	7.49	0.233
		Outdoor	642	80	8.03	
	Control	Indoor	568	40	14.2	< 0.0001
		Outdoor	313	40	7.83	

*Abbreviations*: HBR, human biting rate; b/p/n, bite/person/night

### Impact of IRS on SI and EIR of *An. gambiae* (*s.l.*)

A total of 8603 head-thoraces of *An. gambiae* (*s.l*.) were analyzed by ELISA CSP over the study period. After the ELISA CSP tests, a mean SI of 8.4% (95% CI: 7.25–9.63%; 181 positive samples out of 2158 tested) was obtained before IRS (June–October 2016) compared to 1.2% (95% CI: 0.8–1.8%; 26 positive samples out of 2122 tested) after the first round of IRS (June–October 2017), corresponding to a reduction rate of 85.71% (*χ*^2^ = 117.69, *df* = 1, *P* < 0.0001). Similarly, an 87.27% reduction in EIR was observed after the first round of IRS (2.7 infective bites/person/month) as compared to the pre-intervention period (21.21 ib/p/month) in Alibori and Donga (*RR* = 7.83 (95% CI: 5.17–12.31), *P* < 0.0001) (Table 5).

**Table 5:** Human-biting rate, sporozoite index and entomological inoculation rate in Alibori and Donga regions (IRS areas) before and after the first round of IRS 2017

Region	Variable	Before IRS (June–October 2016)	After 1st round of IRS (June–October 2017)	Reduction (%)
IRS areas (Alibori, Donga)	HBR/night	8.43	7.37	–
	SI (%)	8.4	1.2	85.71
	EIR (ib/person/night)	0.707	0.09	–
	EIR (ib/person/month)	21.21	2.7	87.27

*Abbreviations*: SI, sporozoite index; HBR, human-biting rate; EIR, entomological inoculating rate; ib, infected bite

This reduction in the SI and EIR of *An. gambiae* (*s.l*.) was also observed in the treated areas as compared to the control ones during the efficacy period of Actellic 300 CS in 2017 and 2018. Indeed, after the first round of IRS (June–September 2017), a mean SI of 0.7% (95% CI: 0.36–1.2%; 12 positive samples out of the 1697 tested) was obtained in the treated areas compared to 3.7% (95% CI: 2.7–5.01%; 42 positive samples out of 1124 tested) in the control areas (*χ*^2^ = 31.45, *df* = 1, *P* < 0.0001) (Table 6). During the same period, a person received a mean 12.11 ib/month in control areas against 1.6 ib/month in the treated areas (*RR* = 7.53 (95% CI: 3.89–12.73), *P* < 0.0001), which equals to an 86.78% reduction in malaria transmission. Moreover, the mosquitoes tested after the second IRS round (June–August 2018) showed a mean SI of 0.64% (95% CI: 0.27–1.2%; 8 positive samples out of 1241 tested) in the treated areas against 3% (95% CI: 1.9–4.2%; 26 positive samples out of 881 tested) in the control areas (*χ*^2^ = 15.954, *df* = 1, *P* < 0.0001) (Table 6). During the same period a significant reduction (84.61%) in the EIR in the treated areas (1.5 ib/p/month) was observed compared to the control areas (9.37 ib/p/month) (*RR* = 6.25 (95% CI: 2.73–16.03), *P* < 0.0001) (Table 6).

**Table 6:** Human-biting rate, sporozoite index and entomological inoculation rate in IRS and control areas

Variable	After 1st IRS campaign (June–September 2017)			After 2nd IRS campaign (June–August 2018)		
	IRS areas (Alibori–Donga)	Control areas (Bembereke–Kouande)	Reduction (%)	IRS areas (Alibori–Donga)	Control areas (Bembereke–Kouande)	Reduction (%)
HBR/night	7.56	10.81	–	7.76	11.01	–
SI (%)	0.71	3.7	80.81	0.6	3	80
EIR (ib/person/night)	0.053	0.403	–	0.05	0.325	–
EIR (ib/person/month)	1.6	12.11	86.78	1.5	9.75	84.61

*Abbreviations*: SI, sporozoite index; HBR, human-biting rate; EIR, entomological inoculation rate; ib, infective bite

**Fig. 5:**
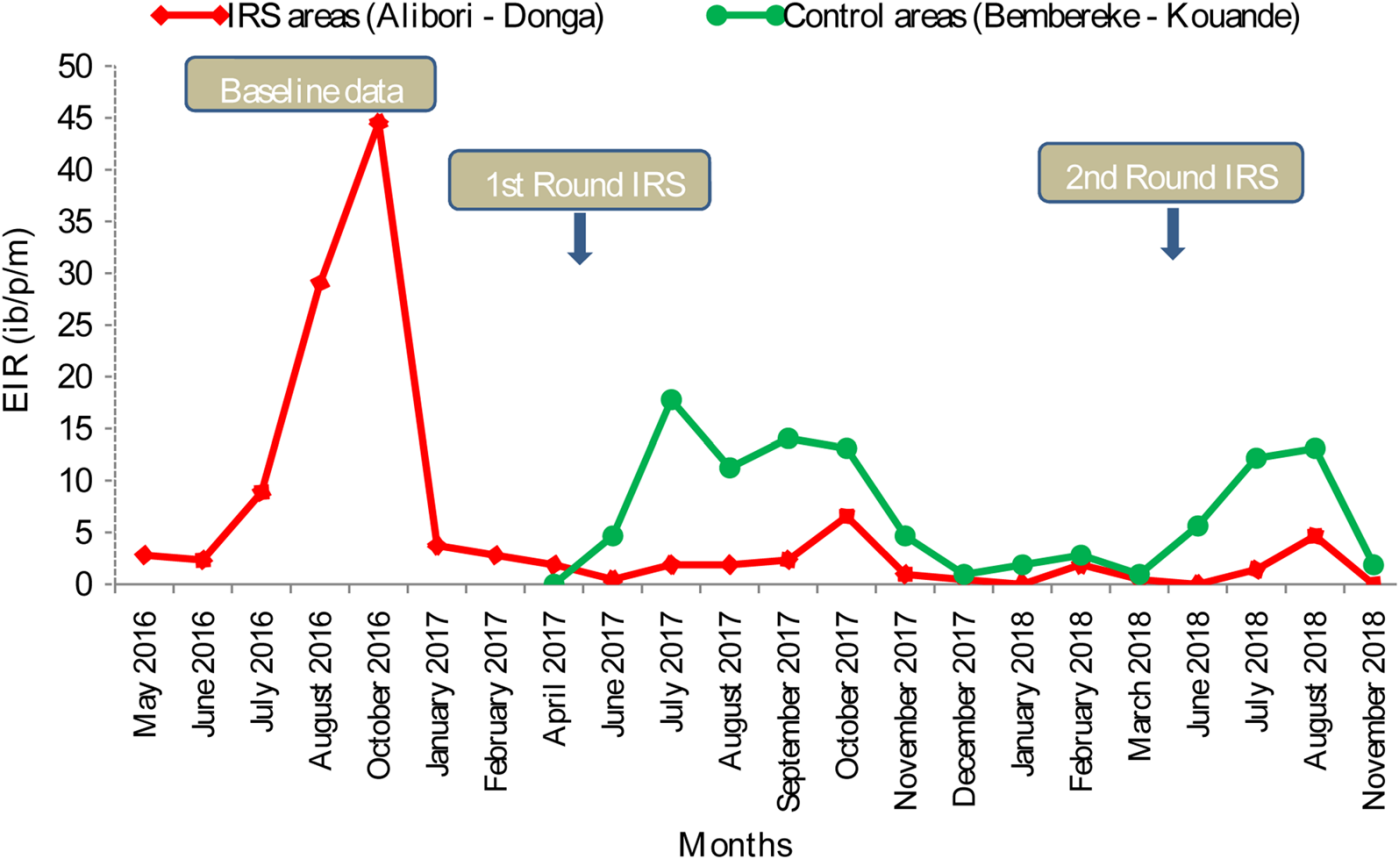
Dynamics of EIR before and after IRS campaigns in treated and control areas from May 2016 to November 2018

Figure 5 shows the dynamics of EIR from May 2016 to November 2018. The lowest EIRs were observed during the dry periods (January 2017 to April 2017 and November 2017 to March 2018) in both treated and control areas. After IRS implementation, lower monthly EIRs were observed in the treated areas compared to the control areas between June and October 2017 and 2018, which equals to 4 months of impact each year (Fig. 5).

### Impact of IRS on IVD in treated and control areas

Table 7 shows the mean indoor density of *An. gambiae* (*s.l*.) before IRS and during Actellic 300 CS efficacy periods. Mean densities of 1.64 *An. gambiae* (*s.l*.) and 0.41 *An. gambiae* (*s.l*.) per room were recorded before (June–October 2016) and after (June–October 2017) IRS in Alibori and Donga, respectively, equating to a 75% reduction (*RR* = 4.04 (95% CI: 3.53–4.64), *P* < 0.0001) (Table 7). In addition, after the first IRS campaign (June–September 2017), 1.53 *An. gambiae* (*s.l*.) per room was recorded in control areas compared to 0.38 *An. gambiae* (*s.l*.) in treated areas, equating to a reduction of 75.16% (*RR* = 4.03 (95% CI: 3.34–4.88), *P* < 0.0001) (Table 7). The trend was the same after the second round of IRS (June–August 2018) where a significant reduction of 72.72% in mean indoor density of *An. gambiae* (*s.l*.) in the treated areas (0.48 *An. gambiae* (*s.l*.) per room) compared to the control areas (1.76 *An. gambiae* (*s.l*.) per room) was observed (*RR* = 3.64 (95% CI: 2.84–4.67), *P* < 0.0001) (Table 7).

**Table 7:** Indoor density of *An. gambiae* (*s.l*.) in IRS and control areas

Period	Area	No. of rooms	No. collected	Density/room	*P*-value
Before IRS (June–October 2016)	Baseline	610	1001	1.64	< 0.0001
After 1st round of IRS (June–October 2017)	After IRS areas	673	273	0.41	
After 1st round of IRS (June–September 2017)	After IRS areas	551	208	0.38	< 0.0001
	Control areas	160	244	1.53	
After 2nd round of IRS (June–August 2018)	After IRS areas	242	117	0.48	< 0.0001
	Control areas	88	155	1.76	

### Impact of IRS on blood-feeding rate of *An. gambiae* (*s.l.*) in treated districts

Overall, blood-feeding decreased significantly after IRS intervention in the study regions. Prior to the intervention (June–October 2016), the blood-feeding rate of *An. gambiae* (*s.l*.) was 96.20%. After the first round of IRS (June–October 2017), this rate decreased to 63.37% in the treated areas, representing a reduction rate of 34.12% (*χ*^2^ = 236.03, *df* = 1, *P* < 0.0001) (Table 8). Similarly, a 22.6% reduction in the blood-feeding rate of *An. gambiae* (*s.l*.) was also observed in the treated areas (62.5%) compared to the control areas (80.74%) during the Actellic 300 CS persistence period (June–September 2017) (*χ*^2^ = 17.76, *df* = 1, *P* < 0.0001) (Table 8).

**Table 8:** Blood-feeding rates in *An. gambiae* (*s.l.*) in treated and control areas

Period	Area	No. collected	Unfed	Fed	Gravid	Half-gravid	Blood-feeding rate %	*P*-value
Before IRS: June–October 2016	IRS areas	1001	26	922	12	41	96.20	< 0.0001
After 1st round of IRS: June–October 2017		273	99	171	1	2	63.37	
After 1st round of IRS: June–September 2017	IRS areas	208	78	129	0	1	62.5	< 0.0001
	Control areas	244	16	187	31	10	80.74	

## Discussion

The persistence of an insecticide applied to walls is one of the key indicators for determining the effectiveness of IRS [29, 30]. In the Alibori and Donga regions, the persistence of Actellic 300 CS used for the two IRS campaigns was 4–5 months (May–September/October). These results corroborate those obtained by Chanda et al. [31] in Zambia and Tchicaya et al. [32] in Mbe (Côte d’Ivoire) who observed a residual efficacy of Actellic 300 CS of 4–5 months on mud and cement walls. However, persistence of Actellic 300 CS observed in this study is lower than that observed in Zanzibar (8 months) by Haji et al. [33] for the same product. These differences in community persistence of Actellic 300CS from one country to another could be due to the influence of several variable factors such as, the environmental conditions (temperature, relative humidity, exposure to ultraviolet rays) [34–38], the composition and characteristics (porosity and pH) of treated walls [39–41] and/or the human interferences with treated surfaces (washing of treated walls) [42].

Two malaria vectors have been encountered in treated and control areas, *An. gambiae* (*s.l*.) (98.05%), which represents the most predominant species, followed by *An. funestus* (1.59%). These data corroborate previous studies performed by Gnanguenon et al. [17] in Kandi (northeast Benin) and Aikpon et al. [43] in Atacora, a region neighboring Donga (northwest Benin). Molecular identification of the sibling species of the *An. gambiae* (*s.l.*) complex revealed that *An. gambiae* (65.60%), *An. coluzzii* (33.42%) and *An. arabiensis* (0.98%) live in sympatry. The predominance of *An. gambiae* in the study area, particularly during rainy season, confirms that dry savannah areas where many temporary breeding sites are formed after rains, are conducive to the development this species. This was highlighted by previous studies carried out in similar bio-ecological areas in Nigeria [44], Cameroon [45] and Burkina Faso [46]. As previously found by Simard et al. [47] and Kudom et al. [48], the predominance of *An. coluzzii* in the dry season during this study could be due to the presence of permanent and semi-permanent breeding sites such as dams and, watering places that are the major larval habitats during this period. Deforestation and increasingly long dry seasons in the study area, are suspected to be unfavorable conditions for the development of *An. arabiensis*, as reflected by the low number recorded in the present study (*n* = 27).

Overall, after the first round of IRS, significantly lower density, SI, blood-feeding and parity rates were observed compared to the pre-intervention period. Similarly, after the first and second rounds of IRS, levels of these indicators were significantly lower in treated areas compared to the control ones. Indeed, treated areas had probably become difficult to live in for the *Anopheles* vectors that fled treated houses. This observation is highlighted here by the low indoor resting density and the more pronounced outdoor biting rate of *An. gambiae* (*s.l.*) which were observed post-IRS intervention in the targeted localities. Thus, only a small proportion of vectors could succeed in taking their blood meals inside treated houses as observed by Sy et al. [49] in west-central Senegal. The reduced blood-feeding rate of the vectors had certainly induced a lower parity rate in treated areas since success in taking a blood meal conditions the maturation of ovaries [50]. In addition, the lethal effect of Actellic 300 CS might not have favored incubation of parasite in vectors before they died, resulting in their low infectivity in the treated areas compared to the control areas. These results confirm the findings by Coleman et al. [51] in northern Ghana and Mashauri et al. [52] in Lake Victoria basin of Tanzania, who also described a significant decrease in the SI of *An. gambiae* (*s.l.*) after IRS with Actellic 300 CS.

Before intervention, the EIR of *An. gambiae* (*s.l*.) was 21.21 ib/p/month in Alibori and Donga. However, after the first round of IRS (June–October 2017), a substantial decrease to 2.7 ib/person/month was detected, which represents a reduction of 87.27%. The high decrease in EIR in sprayed areas compared to the control areas (86.78% in 2017 and 84.61% in 2018) could be due to a high IRS coverage in treated areas (more than 91% IRS coverage in 2017 and 92% in 2018). Indeed, similar results were obtained in Zambia [31], Zimbabwe [53], Tanzania [52] and Uganda [54] where more than 85% IRS coverage with Actellic 300 CS was achieved. In parallel, the results of an epidemiological study conducted in 2017 after IRS revealed that the decrease of EIR was accompanied by an 8% decrease of incidence among children under five years of age in the treated area of Donga [55]. EIR reduction (86.78% in 2017 and 84.61% in 2018) in our study area is lower than that previously observed by Akogbéto et al. [11] (94.4% in Oueme, southern Benin), Aikpon et al. [12] (99.24% in Atacora, northwest Benin) and Sy et al. [49] (92.59% in central-west Senegal).

Although blood-feeding rates and SI of *Anopheles* were significantly lower in the treated areas compared to the control areas, they remained considerable in these areas. This highlights the need to support IRS campaigns with information, education and communication campaigns to aware the population on the necessity of sleeping under LLINs, even in sprayed houses to avoid bites of mosquitoes which do not succeed in resting on walls due to the insecticide effect.

Currently, pirimiphos-methyl CS is the only product used in IRS in Benin due to the emergence of resistance of *An. gambiae* (*s.l*.) to bendiocarb and pyrethroids [14]. Since resistance is a dynamic phenomenon, its emergence to organophosphates over time cannot be ruled out. Considering this, the use of new generation insecticides such as SumiShield^®^ 50WG and Fludora^®^ Fusion which have a persistence of approximately 8 to 10 months [56, 57] and which have been recently approved by WHO, may be considered. This will allow covering the entire duration of malaria transmission (approximately 6 months) in the two surveyed regions.

The present study has some limitations. Indeed, the discontinuation of the cone bioassays in September 2018 when Actellic 300CS was still effective meant the exact persistence duration of the product could not be determined. Moreover, the mosquito collections which started at 21:00 h did not allow collecting information on early mosquito biting in the evening.

## Conclusions

The reduction of key entomological indicators of malaria transmission in the treated regions shows the positive impact of the IRS programme. However, this strategy must be complemented with a high use of LLINs for greater effectiveness in vector control.

## Data Availability

Data supporting the conclusions of this article are included within the article. The data used and/or analyzed in this study are available from the corresponding author upon reasonable request.

## Abbreviations

IRS: indoor residual spraying
PMI: Presidentʼs Malaria Initiative
USAID: United States Agency for International Development
WHO: World Health Organization
LLIN: long-lasting insecticidal nets
NMCP: National Malaria Control Programme
EIR: entomological inoculation rate
HBR: human-biting rate
ELISA: enzyme-linked immunosorbent assay
PCR: polymerase chain reaction
CSP: circumsporozoite protein
HLC: human landing catch
SI: sporozoite index
PSC: pyrethrum spray catch
IVD: indoor vector density
WG: water dispersible granules
CS: micro-encapsulated formulation
CREC: Centre de Recherche Entomologique de Cotonou
